# An Ionic Liquid Ablation Agent for Local Ablation and Immune Activation in Pancreatic Cancer

**DOI:** 10.1002/advs.202206756

**Published:** 2023-01-25

**Authors:** Junming Huang, Meng Wang, Fu Zhang, Shiyi Shao, Zhuo Yao, Xinyu Zhao, Qida Hu, Tingbo Liang

**Affiliations:** ^1^ Department of Hepatobiliary and Pancreatic Surgery First Affiliated Hospital Zhejiang University School of Medicine Hangzhou 310006 P. R. China; ^2^ Zhejiang Provincial Key Laboratory of Pancreatic Disease Hangzhou 310003 P. R. China; ^3^ Innovation Center for the Study of Pancreatic Diseases Hangzhou 310003 P. R. China; ^4^ Zhejiang Provincial Clinical Research Center for the Study of Hepatobiliary & Pancreatic Diseases Hangzhou 310003 P. R. China; ^5^ Cancer Center Zhejiang University Hangzhou 310058 P. R. China; ^6^ Research Center for Healthcare Data Science Zhejiang Lab Hangzhou 310003 P. R. China

**Keywords:** immunotherapy, ionic liquid, pancreatic cancer, tumor ablation

## Abstract

Pancreatic ductal adenocarcinoma rapidly acquires resistance to chemotherapy, remaining a fatal disease. Immunotherapy is one of the breakthroughs in cancer treatment, which includes immune checkpoint inhibitors, chimeric antigen receptor T‐cell immunotherapy, and neoantigen vaccines. However, immunotherapy has not achieved satisfactory results in the treatment of pancreatic cancer. Immunogenic death comprises proinflammatory cell death, which provides a way to enhance tumor immunogenicity and promote an immune response in solid tumors. Herein, an ionic liquid ablation agent (LAA), synthesized from choline and geranic acid, which triggers necrosis‐induced immunotherapy by remodeling an immunosuppressive “cold” tumor to an immune activated “hot” tumor is described. The results indicate that LAA‐treated tumor cells can enhance immunogenicity, inducing dendritic cell maturation, macrophage M1 polarization, and cytotoxic T lymphocyte infiltration. The results of the present study provide a novel strategy for solid tumor immunotherapy.

## Graphical abstract

An ionic liquid (IL) agent comprising bicarbonate and geranic acid is engineered for ablation treatment of pancreatic cancer, providing a new method, IL ablation, to initiate and amplify the immune response against immune‐suppressive solid tumors, providing a potential solution to immunotherapy inefficiency.

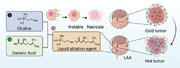

## 1 Introduction

Pancreatic carcinoma, featuring early metastasis and poor survival, is likely to become the second leading cause of cancer‐related death in the next decade.^[^ ^1^ ^]^ Curative treatments, such as surgical resection, could be applied for patients with early‐stage disease; however, they only comprise 15–20% of the whole pancreatic carcinoma population.^[^ ^2^ ^]^ The other patients with pancreatic cancer receive systemic chemotherapy, which has an unsatisfactory response rate of 5–20% and a small chance of transforming the tumor to a resectable state.^[^ ^3^, ^4^ ^]^ Another treatment option for inoperable pancreatic cancer is immune checkpoint blockade (ICB); however, ICB has shown limited efficiency in several recent clinical trials.^[^ ^5^, ^6^ ^]^ The immunosuppressive microenvironment and desmoplastic stroma in pancreatic carcinoma might contribute to the lack of therapeutic efficiency of systemic therapy.^[^ ^7^, ^8^, ^9^ ^]^ Therefore, there is an urgent need to develop strategies that simultaneously induce tumor regression and regulate the immune microenvironment, to improve the outcome of current conventional therapies.

Locoregional treatments, including interventional embolization and ablative techniques, have proven to be beneficial for a highly selected group of patients with oligometastatic pancreatic carcinoma,^[^ ^10^ ^]^ and might represent a breakthrough strategy to bridge tumor inhibition and focal immune regulation. Contemporary ablation therapies use thermal or electrical energy, or focused radiation beams, to achieve focal destruction of tumor tissue.^[^ ^11^, ^12^ ^]^ However, because of the unique anatomy of the pancreas, being adjacent to several key vessels and delicate intestines, energy‐based ablation therapies might cause damage to peripancreatic tissues. In addition, these approaches rarely alter the desmoplasia‐associated “cold” tumor status, which is mainly characterized by the absence of infiltrating immune cells.

Nonenergy ablation, i.e., chemical ablation, is a possible solution to avoid energy‐related injuries. Percutaneous ethanol injection, which has been widely used for the ablation of hepatocellular carcinoma, thyroid cancer, and many types of benign tumors, induces protein denaturation leading to coagulative necrosis, thrombosis of small vessels, and formation of fibrotic and granulomatous tissue. Ethanol ablation could also be applied to pancreatic tumors via endosonography‐guided injection; however, ethanol diffuses throughout the tumor and the surrounding parenchyma, resulting in inefficient intratumoral diffusion and leading to residual tumor and high recurrence rates.^[^ ^13^, ^14^ ^]^ Ionic liquid (IL), a salt in the liquid state, might be the ideal candidate as an ablative agent.^[^ ^15^, ^16^ ^]^ It is hypothesized that hydrophobic anions in the IL would interact with tumor cell membranes, causing membrane instability and enhancing its diffusion through the tumor.^[^ ^17^, ^18^ ^]^ IL has been applied for chemical ablation in liver and kidney cancer, in which it outperformed ethanol in terms of its ablative effect.^[^ ^15^ ^]^ The strong diffusion ability of IL is also used to facilitate the delivery of drug cargos through the physical barriers of the skin and the intestinal mucosa.^[^ ^19^, ^20^ ^]^

**Scheme 1:**
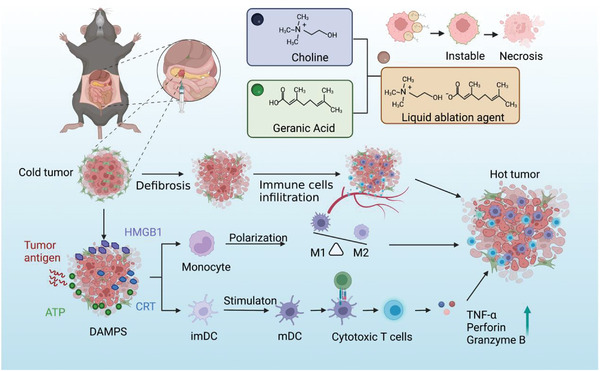
Schematic illustration of IL‐induced antitumor immunotherapy.

A recent study showed that IL could ameliorate the poor ablative effect in solid dense tumors.^[^ ^15^ ^]^ Further pathological examination demonstrated increased numbers of infiltrating CD8^+^ T cells intratumorally after IL injection, suggesting that the enhanced efficiency is associated with an altered immune response.^[^ ^15^ ^]^ However, the mechanism of immune microenvironment remodeling after IL ablation in malignancies, specifically in dense tumors like pancreatic cancer, remains unclear. Herein, we synthesized an IL agent comprising bicarbonate and geranic acid for ablation treatment of pancreatic cancer, evaluated the immune response to IL ablation in immunosuppressive pancreatic tumors, and investigated the corresponding underlying mechanism (**Scheme** **1**).

## 2 Results

### 2.1 Liquid Ablation Agent (LAA) Effectively Ablates Pancreatic Cancer

**Figure 1:**
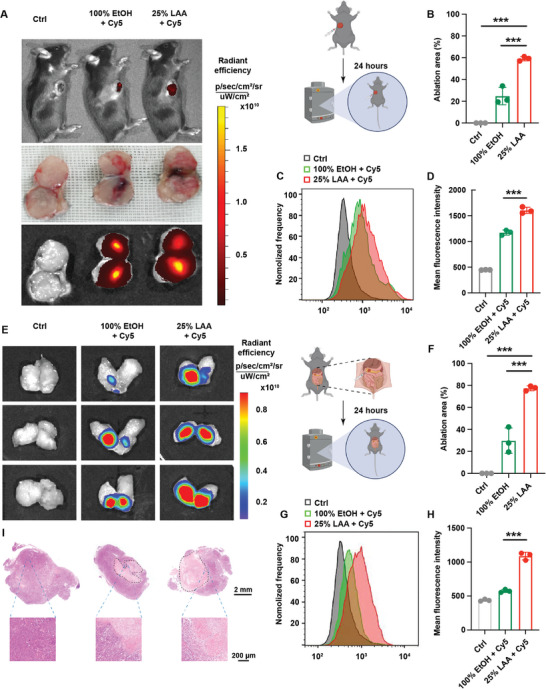
LAA effectively ablates solid tumors. A) Representative images of in vivo Cy5 fluorescence distribution, with bright‐field photos and fluorescence images of the tumor cross‐sections in the subcutaneous pancreatic tumor at 24 h after intratumoral injection of PBS (control), saline plus Cy5, EtOH plus Cy5, or LAA plus Cy5. B) Comparison of the ablated areas in the treated tumors. C) Representative Cy5 fluorescence patterns and D) the quantitative comparison of Cy5 fluorescence intensity in the tumor samples after the indicated treatments using flow cytometry analyses. E) Representative images of in vivo Cy5 fluorescence distribution in the mice with orthotopic pancreatic cancer at 24 h after intratumoral injection of saline plus Cy5, EtOH plus Cy5, or LAA plus Cy5. F) Comparison of the ablated areas in the treated tumors. G) Representative Cy5 fluorescence patterns and H) the quantitative comparison of Cy5 fluorescence intensity in the tumor samples after the indicated treatments using flow cytometry analyses. I) Histological study of the treated tumors showing necrotic areas after ablation (marked by dashed lines). *p < 0.05, ***p < 0.001, *n* = 3.

LAA was synthesized via an ionic metathesis reaction between choline bicarbonate and geranic acid at a molar ratio of 1:1, creating the IL, as previously reported (Figure S1, Supporting Information).^[^ ^15^ ^]^ The fluorescent dye Cyanine5 (Cy5) was mixed with different concentrations of LAA solution, ranging from 5% to 50%, to investigate its concentration‐dependent diffusion ability. Among the LAA solutions, 25% LAA exhibited the strongest local diffusion ability compared with the LAA solutions at 5%, 10%, and 50%, indicating that 25% was the optimal concentration for potential ablative therapy in pancreatic cancer, which was used for subsequent in vitro experiments (Figure S2, Supporting Information). Absolute ethanol (100% EtOH) is an ablative agent commonly used for tumor treatment in clinical practice.^[^ ^21^ ^]^ LAA displayed a greater diffusion‐based ablative ability in subcutaneous xenograft pancreatic cancer in C57BL/6 mice, with an average ablated area of 559.0 ± 35.4 mm^2^ (mean ± SD, *n* = 3), which was significantly larger than that after absolute ethanol treatment (140 ± 6.6 mm^2^; *p* < 0.001, one‐way analysis of variance (ANOVA) followed by Tukey's test) or saline (0 ± 0 mm^2^; *p* < 0.001, Tukey's test) (**Figure** **1**A,B), which was further confirmed by assessing Cy5‐positive fluorescence intensity via flow cytometry analyses (Figure 1C,D). Furthermore, Cy5‐labeled LAA revealed a similar ablative ability in orthotopic pancreatic cancer in C57BL/6 mice, with a larger ablated area of 641.3 ± 173.7 mm^2^ (mean ± SD, *n* = 3) compared with that of absolute ethanol (132.0 ± 32.0 mm^2^; *p* = 0.002, Tukey's test) (Figure 1E,F). Flow cytometry verification showed a twofold higher Cy5 positivity rate in LAA‐treated tumors than in ethanol‐treated tumors (Figure 1G,H). Pathological analyses of the ablated orthotopic tumors showed ablation‐induced necrosis, with irregular diffusion patterns after absolute ethanol ablation and relatively uniform patterns after LAA treatment, confirming the better ablation efficacy of LAA compared with absolute ethanol (Figure 1I). Accordingly, these results suggested that 25% LAA outperformed absolute ethanol in its ablative efficacy in pancreatic cancer.

### 2.2 LAA Ablation Enhances Intratumoral Permeability

**Figure 2:**
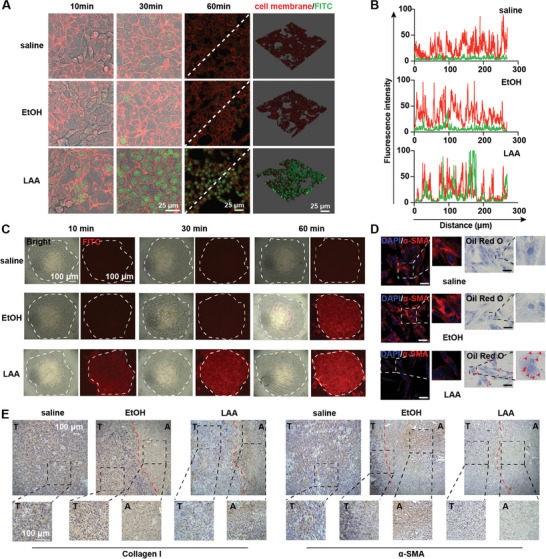
LAA ablation facilitates intratumoral permeability. A) Representative confocal microscopy images of KPC cells at 10, 30, and 60 min after treatment with free FITC (green), EtOH plus FITC, or LAA plus FITC. The cell membrane was stained using CellMask (red). The scale bar represents 25 µm. B) Cross‐sectional analyses of fluorescence intensity of the cell membrane (red) and FITC (green) on the white dashed line. C) The KPC spheroids treated with free FITC (red), EtOH plus FITC, or LAA plus FITC. The scale bars represent 100 µm. D) Representative confocal microscopy images of *α*‐SMA expression (red) and lipid droplets (indicated by red triangles) in pancreatic stellate cells (PSCs) treated with saline, EtOH, or LAA. The nuclei were stained with 4′,6‐diamidino‐2‐phenylindole (DAPI, blue). The scale bars represent 50 µm. E) Immunohistochemistry analyses of collagen I and *α*‐SMA expression levels in KPC tumors treated with saline, EtOH, or LAA. The ablated area and the tumor area are marked by the letters A and T, respectively. The scale bars represent 100 µm.

To validate whether LAA was able to penetrate rapidly into the deep tissue of pancreatic cancer, we investigated its permeability at both the cytological level and in terms of spatial dimensions. The concentrations of EtOH and LAA were reduced to 1% to avoid rapid cell death, which was suitable for permeability studies. For the cytological study, KPC cells incubated with fluorescein isothiocyanate (FITC) dye in saline, 1% EtOH (EtOH/FITC), or 1% LAA (LAA/FITC) were monitored using confocal laser scanning microscopy to determine the impact of LAA on intracellular trafficking. The results illustrated a higher and more sustained FITC fluorescence in the nuclei of KPC cells exposed to LAA/FITC compared with that in cells treated with free FITC or EtOH/FITC (**Figure** **2**A,B). This suggested that LAA facilitated the internalization and cytoplasmic trafficking of solute substances. In the spatial study, KPC spheroids were introduced to evaluate the intercellular penetration ability of FITC with the aid of LAA from a 3D perspective. Time‐dependent tracking of FITC fluorescence showed that the LAA solvent allowed cargo substances to rapidly accumulate in the interior part of the spheroids (Figure 2C). In contrast, EtOH induced relatively slow accumulation of FITC in the spheroids, while free FITC was not able to penetrate the spheroids. These results demonstrated that LAA enhanced the intercellular penetration of the cargo substances.

However, the pancreatic cancer microenvironment, with distinct desmoplastic stroma composed of extracellular matrix (ECM) and cancer‐associated fibroblasts (CAFs), which hinders intratumoral drug diffusion, is more complex than KPC spheroids. CAFs derived from activated pancreatic stellate cells (PSCs) produced large amounts of ECM, serving as a barrier for effective drug delivery to tumor cells.^[^ ^22^ ^]^ Therefore, we investigated how LAA altered PSC activation and ECM density in pancreatic cancer. LAA, in comparison with EtOH, not only enhanced the permeability of PSCs (Figure S3, Supporting Information), but also markedly decreased the expression of *α*‐smooth muscle actin (*α*‐SMA) and increased the number of vitamin A‐containing lipid droplets in PSCs (Figure 2D), indicating that LAA was capable of inhibiting PSC activation. Inspired by LAA's impact on PSC activation, we further investigated its role in regulating CAFs and the ECM in the ablated pancreatic cancer tissue. An immunohistochemical study demonstrated significant reductions in *α*‐SMA and collagen I levels, both in the ablated area and in the residual tumor tissue (Figure 2E), suggesting that LAA ablation depleted CAFs and ECM in pancreatic cancer, which would contribute to decreasing the stromal desmoplasia and enhancing intratumoral permeability.

### 2.3 LAA Suppresses Pancreatic Cancer In Vitro

**Figure 3:**
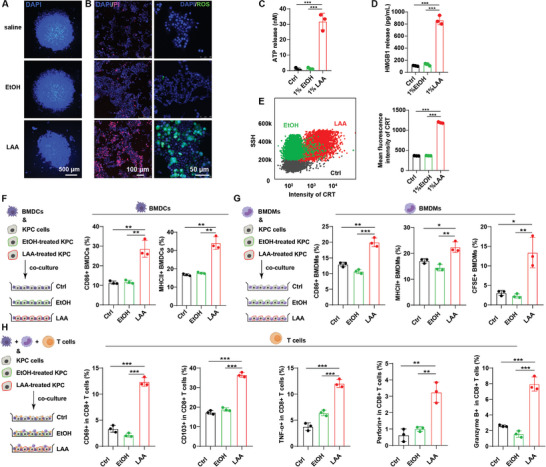
LAA suppresses pancreatic cancer in vitro. A) KPC cell spheroids treated with saline, EtOH, or LAA for 24 h. The nuclei are stained with DAPI (blue). The scale bars represent 500 µm. B) PI (in pink) and reactive oxygen species (ROS, in green) staining of the KPC cells incubated with saline, EtOH, or LAA. The scale bars represent 100 µm. C) ATP release and D) HMGB1 release in the KPC cells treated with saline, EtOH, or LAA. E) Membrane calreticulin (CRT) expression in the KPC cells treated with saline, EtOH, or LAA using flow cytometry analyses. F) CD86 and MHC II levels in BMDCs co‐cultured with the treated KPC cells. G) CD86, MHC II, and CFSE levels in BMDMs co‐cultured with the treated KPC cells. H) CD69, CD103, TNF‐*α*, perforin, and granzyme B levels in CD8^+^ T cells co‐cultured with BMDCs, BMDMs, and the treated KPC cells. *p < 0.05, **p < 0.01, ***p < 0.001, *n* = 3.

To examine whether LAA exerted cytotoxicity toward pancreatic cancer cells, we performed in vitro experiments in KPC cells and spheroids treated with saline, EtOH, or LAA. The stability of the KPC spheroids was partially destroyed when exposed to LAA, but was maintained when exposed to saline or EtOH (**Figure** **3**A), implying that LAA outperformed EtOH in terms of cytotoxicity. The cytotoxicity of LAA was further measured by the fluorescence intensity of propidium iodide (PI) and the production of reactive oxygen species (ROS), which correlated with the extent of cell death. PI fluorescence intensity and ROS levels were both elevated in KPC cells treated with LAA compared with those treated with phosphate‐buffered saline (PBS) or EtOH (Figure 3B), confirming that LAA enhanced cytotoxicity. Intensive necrosis‐related death frequently causes immunogenic cell death (ICD);^[^ ^23^, ^24^ ^]^ therefore, we evaluated damage‐associated molecular patterns (DAMPs), which correspond to ICD activities, after LAA treatment, including adenosine triphosphate (ATP) secretion, high mobility group protein B1 (HMGB1) release, and calreticulin (CRT) exposure.^[^ ^25^ ^]^ The LAA‐treated KPC cells showed a 41.5‐fold higher level of ATP secretion and an 8.2‐fold higher level of HMGB1 release than the EtOH‐treated cells (Figure 3C,D). Meanwhile, flow cytometry detected elevated expression of calreticulin on the surface of the LAA‐treated cells, which was three times higher than that of the EtOH‐ or saline‐treated cells (Figure 3E). Thus, LAA effectively regulated DAMPs, which indicated a major contributing role of ICD to LAA‐related death in the ablated pancreatic cancer.

### 2.4 LAA Activates Antitumor Immunity In Vitro

Based on the rationale that ICD induces activation of antitumor immunity, we next explored the immune responses to LAA stimuli in vitro using a co‐culture system consisting of the treated KPC cells and bone marrow‐derived dendritic cells (BMDCs), bone marrow‐derived macrophages (BMDMs), or T lymphocytes, to mimic the tumor microenvironment (TME).

The co‐culture systems of KPC cells and BMDCs or BMDMs were first established to evaluate the effect of LAA treatment on dendritic cells (DCs) or macrophages. CD86 and major histocompatibility group (MHC) II are the distinctive markers of DC maturation and macrophage polarization toward the proinflammatory M1 phenotype.^[^ ^26^ ^]^ Co‐culture with LAA‐treated KPC cells induced 1.5‐fold higher levels of CD86 and MHC II on BMDCs and twofold higher levels on BMDMs, in comparison with the saline and EtOH groups (Figure 3F,G). Their representative cytometry patterns are shown in Figure S4 (Supporting Information). These results suggested that LAA, unlike the conventional ablative agent EtOH, exhibited greater efficacy in facilitating DC maturation and M1 macrophage polarization. Additionally, the LAA‐treated KPC cells labeled with carboxyfluorescein succinimidyl ester (CFSE), but not the EtOH‐treated KPC cells, yielded an increased CFSE‐positive proportion in the co‐cultured BMDMs, suggesting that the enhanced antigen‐presenting capacity of the BMDMs in the LAA group allowed for engulfment of more CFSE‐labeled tumor cells (Figure 3G).

The maturation and proliferation of CD8^+^ T cells is another important constituent of antitumoral immunity.^[^ ^26^ ^]^ The impact of LAA‐treated KPC cells on activities of CD8^+^ T cells was then studied in a co‐culture system composed of KPC cells, BMDCs, BMDMs, and spleen‐derived CD8^+^ T cells. CD69 is a classical early marker of T cell activation after stimulation of the T‐cell receptor,^[^ ^27^ ^]^ and CD103 is an integrin marker that is markedly upregulated when T cells infiltrate the tumor, which indirectly indicates simultaneous T cell activation.^[^ ^28^ ^]^ Compared with those in the control group and the EtOH group, the proportions of CD69^+^ and CD103^+^ T cells were both significantly elevated in the LAA group (Figure 3H), which implied that LAA could drive T cell activation in vitro. Their representative cytometry patterns are shown in Figures S5 (Supporting Information). Activated CD8^+^ cytotoxic T‐lymphocytes (CTLs) produce killing cytokines, including tumor necrosis factor alpha (TNF‐*α*), perforin, and granzyme B, which target the tumor cells and trigger apoptosis.^[^ ^29^, ^30^ ^]^ The levels of these secreted cytokines were measured using flow cytometry to verify whether LAA could induce T cell activation in the co‐culture system. As expected, levels of secreted TNF‐*α*, perforin, and granzyme B were all significantly increased in the LAA group, compared with those in the control and EtOH groups (Figure 3H), confirming the substantial role of LAA in generating T cell activation. These results demonstrated that LAA‐treated KPC tumor cells possessed high immunogenicity, which enhanced their recognition by macrophages and DCs, and induced T cell activation, generating an improved immune response against pancreatic cancer.

### 2.5 LAA‐Activated Immune Responses Suppress Tumor Progression

To further evaluate the role of LAA ablative therapy in regulating the immune response, we constructed two tumor‐bearing models, namely, immunodeficient BALB/c nude mice with subcutaneous KPC xenograft tumors and immunocompetent C57BL/6 mice with orthotopic KPC tumors, for comparison of the LAA‐mediated inhibitory effect in pancreatic cancer. The BALB/c nude mice lack a thymus and are thus unable to produce T cells, making them suitable to explore the role of T cells in LAA therapy when compared with C57BL/6 mice possessing a normal immune system.

**Figure 4:**
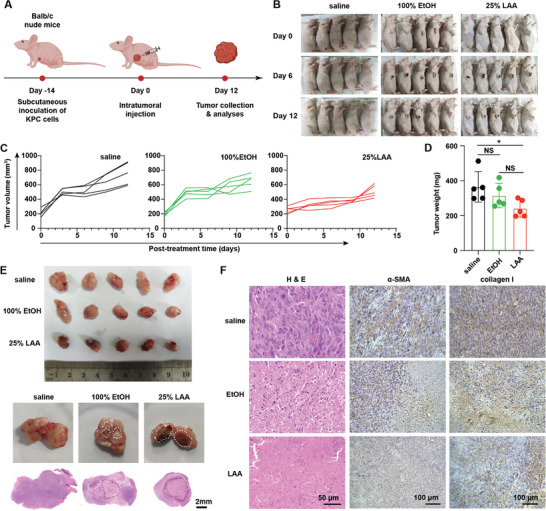
In vivo therapeutic effect of LAA ablation in tumor‐bearing nude mice. A) Schematic of the treatment protocol. B) Images of the subcutaneous KPC tumors treated with saline, EtOH, or LAA. C) Growth curves of the treated tumors. D) Comparison of the final tumor weights. E) Bright‐field images of the excised tumors and the corresponding representative cross‐sectional H&E staining analyses. The necrotic areas are marked with white dashed lines. The scale bars represent 2 mm. F) H&E analyses, and *α*‐SMA and collagen I expression levels assessed using immunohistochemical analyses of the treated KPC tumors. The scale bars represent 50 and 100 µm. NS, not significant; *p < 0.05, *n* = 5.

We first treated the BALB/c mice bearing subcutaneous KPC tumors with intratumoral injection of PBS, 100% EtOH, or 25% LAA (**Figure** **4**A). Compared with the PBS control and 100% EtOH ablation, LAA ablation slowed down tumor growth in the 12 days following ablation therapy (Figure 4B,C). Comparison of the average tumor weight showed that LAA ablation was superior to PBS (*p* = 0.0156, vs PBS; Tukey's test), but no better than EtOH (*p* = 0.1820, vs EtOH; Tukey's test), which suggested that LAA ablation participated in tumor inhibition with limited efficacy (Figure 4D). Further histological study revealed that LAA induced significantly larger ablation‐related necrosis areas compared with those induced by EtOH (LAA, 27.1 ± 2.8 mm^2^; EtOH, 5.5 ± 0.5 mm^2^; *p* = 0.0002, Student's *t* test) (Figure 4E). Interestingly, hematoxylin and eosin (H&E) staining assays also demonstrated that the LAA‐induced necrosis was more uniform and complete compared with that in the EtOH group (Figure 4F). Meanwhile, we also observed reduction in *α*‐SMA expression and loss of collagen I in the LAA‐treated tumors, and a similar, but minor effect, occurred in the EtOH‐treated tumors (Figure 4F), indicating that LAA outperformed EtOH in both ECM inhibition and the focal ablative effect at the histological level.

**Figure 5:**
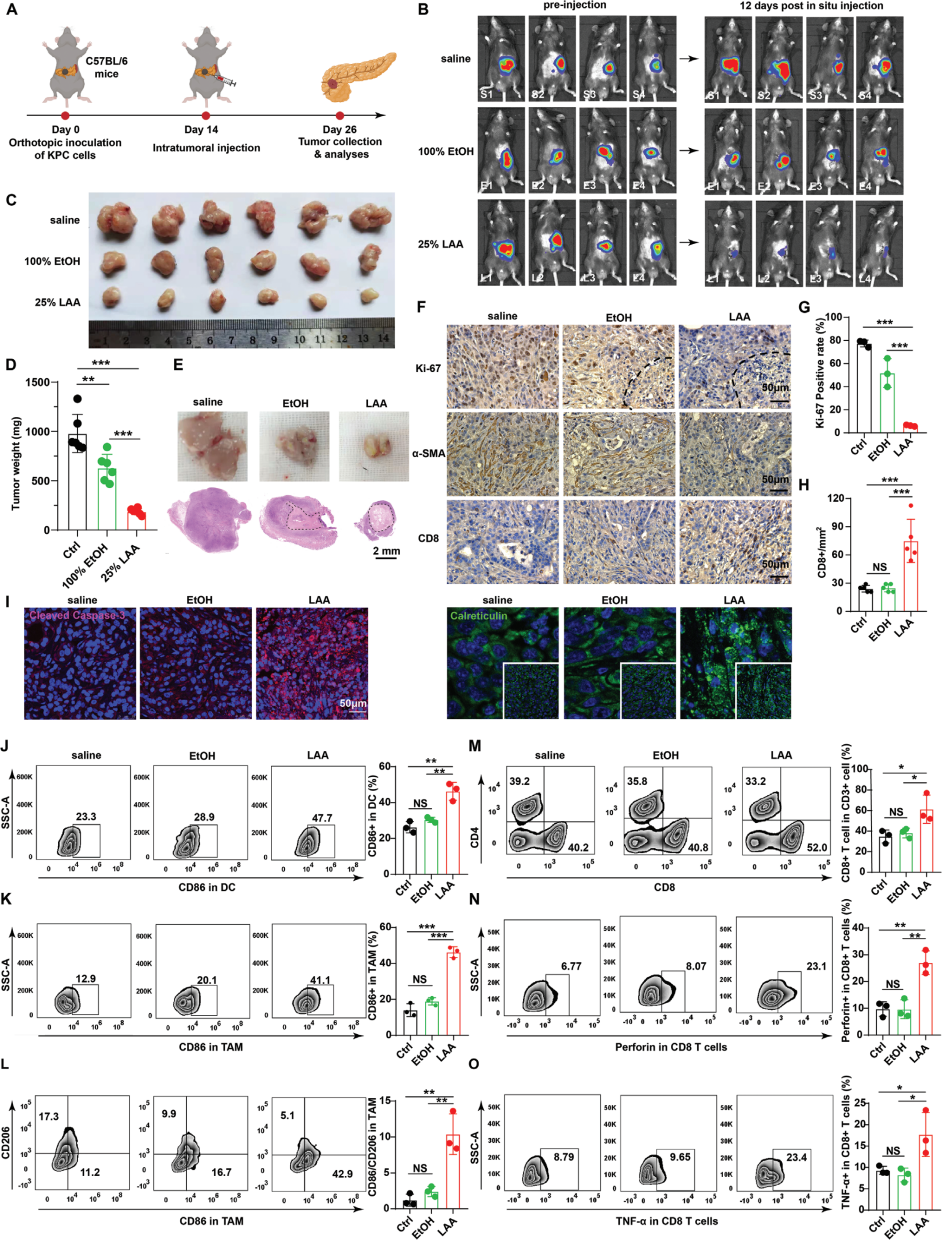
In vivo therapeutic effect of LAA ablation against pancreatic tumors in C57BL/6 mice. A) Schematic of the treatment protocol for the orthotopic KPC^Luc^ pancreatic cancers. B) In vivo luciferase fluorescence in orthotopic pancreatic tumors before and after treatment with saline, EtOH, or LAA. C) Bright‐field images of the excised tumors and the corresponding representative cross‐section analyses. D) Comparison of the final tumor weight (*n* = 6). E) Histological analyses of the tumor cross‐sections with H&E staining. Necrosis is marked using black dashed lines. The scale bar represents 2 mm. F) Ki‐67, *α*‐SMA, and CD8 expression levels determined using immunohistochemical analyses of the treated KPC tumors. The scale bars represent 50 µm. Quantitative analyses of the G) Ki‐67 positive rate (*n* = 3) and the H) CD8^+^ cell count (*n* = 5). I) Expression levels of cleaved Caspase‐3 (red) and calreticulin (green) in the treated tumors. The bars represent 50 µm. J) Intratumoral CD86 expression patterns in dendritic cells (DCs). K) CD86 expression patterns in tumor‐associated macrophages (TAMs). L) CD86 and CD206 expression patterns in TAMs with quantitative comparison of CD86^+^/CD206^+^ ratios. M) CD8 positive expression patterns in CD3^+^ cells. N) Perforin and O) TNF‐*α* expression patterns in CD8^+^ T cells. *p < 0.05, **p < 0.01, ***p < 0.001, *n* = 3.

The imbalance between LAA's ablative effect and tumor inhibitory outcome in the nude mice might be associated with immunodeficiency; therefore, we studied the ablative and inhibitory effects of LAA on the C57BL/6 mice bearing orthotopic fluorescence‐labeled KPC (KPC^Luc^) tumors (**Figure** **5**A). The in vivo florescence monitoring suggested that LAA ablation significantly suppressed tumor growth at 12 days post‐treatment, while treatments with saline or EtOH had low or no efficacy in terms of tumor inhibition (Figure 5B). Comparison of the final tumors demonstrated that LAA ablation surpassed EtOH ablation in terms of the reduction in tumor size (*p* < 0.0001, LAA vs EtOH; Tukey's test) and weight (*p* = 0.0002, LAA vs EtOH; Tukey's test), confirming the advantage of LAA in tumor inhibition (Figure 5C,D and Figure S6, Supporting Information), without any adverse side effects, such as main organ dysfunction or pathological abnormalities (Figures S7 and S8, Supporting Information). Visual inspection and histological analyses both showed that LAA, in comparison to EtOH, resulted in larger ablation‐related necrosis areas and fewer residual tumors (Figure 5E). Meanwhile, immunohistochemistry assays also revealed substantial downregulation of marker of proliferation Ki‐67 expression and reduction of *α*‐SMA after LAA treatment compared with that in the EtOH group, implying that LAA ablation constrained the tumor's proliferative activity and ECM production at the histological level (Figure 5F,G). Therefore, LAA showed a remarkable tumor inhibition ability corresponding to its superior effect of focal ablation on KPC tumors in the immunocompetent mice.

Notably, the immunohistochemistry study of CD8 expression also revealed that LAA ablation, but not EtOH, induced a high proportion of CD8‐positive infiltrating cells compared with that in the control group (Figure 5F,H), indicating a potential role for the immune response in the ablation‐related inhibitory performance. Furthermore, we observed elevated expression of the DAMPs marker, calreticulin, as well as increased expression of the necrotic indicator, cleaved caspase‐3, in LAA‐treated tumors, but not in the saline‐ or EtOH‐treated tumors (Figure 5I), suggesting that LAA‐induced necrosis might correlate with ICD events and associated immune processes.

Inspired by these results, we then explored the underlying immune mechanisms of LAA ablation in tumor inhibition. DCs play a critical role in T cell activation, in which the immune responsiveness is mediated by antigen exposure and antigen‐presenting cell (APC) activation, which correlates with CD86 expression.^[^ ^31^ ^]^ LAA treatment, but not EtOH treatment, increased CD86 expression in DCs in the tumors (*p* = 0.0034, LAA vs EtOH; Tukey's test), indicating that LAA promoted the antigen‐presenting function of DCs (Figure 5J). Another important component in the antitumor immune response, tumor‐associated macrophages (TAMs), also substantially alter immune regulation.^[^ ^32^ ^]^ TAMs, characterized by positive CD11b and F4/80 expression (CD11b^+^ F4/80^+^), are a subpopulation featuring plasticity and heterogeneity, which can be broadly divided into a proinflammatory phenotype (M1‐like, with positive CD86 expression) that suppresses tumors and an anti‐inflammatory phenotype (M2‐like, with positive CD206 expression) that promotes tumors.^[^ ^33^ ^]^ The results showed that LAA treatment, in comparison with EtOH, increased the proportion of CD86‐positive cells (*p* < 0.0001; Tukey's test) and the M1/M2 ratio (*p* = 0.0034; Tukey's test), in the whole TAM population, indicating that LAA regulated TAM polarization toward the M1‐like phenotype (Figure 5K,L and Figure S9, Supporting Information). CD8^+^ CTLs are the main effectors that terminate cancer cells, mainly by secreting cytotoxic cytokines including TNF‐*α* and perforin.^[^ ^34^, ^35^ ^]^ TNF‐*α* and perforin also act as markers of CTL activation. Our results showed that compared with EtOH, LAA significantly increased the amount of CD8^+^ T cells (*p* = 0.0472; Tukey's test) (Figure 5M and Figure S10, Supporting Information), which was consistent with the immunohistochemical study showing CD8 positive infiltrating cells (Figure 5F and Figure S11, Supporting Information), suggesting that LAA ablation induced CTL infiltration in the tumors. In addition, further analyses showed that LAA treatment resulted in elevated proportions of TNF‐*α* ^+^ (*p* = 0.0244, vs EtOH; Tukey's test) and perforin^+^ CTLs (*p* = 0.0026, vs EtOH; Tukey's test) cells, indicating that LAA could not only increase the CTL population, but also enhance CTL activity (Figure 5N,O).

### 2.6 Ablation‐Related Regulation Reverses the Immune‐Suppressive Microenvironment

**Figure 6:**
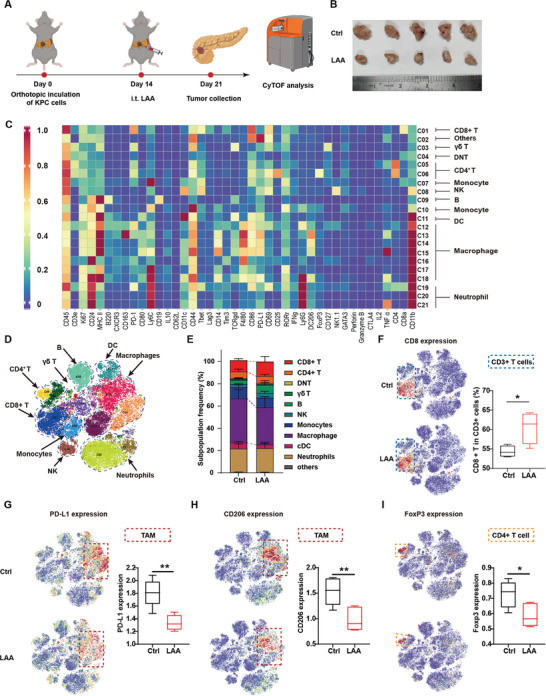
LAA ablation alters the tumor immune microenvironment. A) Schematic of the mass cytometry (CyTOF) analyses protocol for the treated orthotopic pancreatic cancers. B) Bright‐field images of the excised tumors after treatment with saline or LAA. C) A heatmap showing the differential expression of 42 immune markers in the 21 cell clusters by CyTOF. D) A *t*‐distributed stochastic neighbor embedding (tSNE) plot via nonlinear dimensionality reduction showing the immune cell clusters in the LAA‐treated tumors. E) Frequency of the immune cell clusters in the treated tumors. F) Color‐coded tSNE plots showing CD8 expression in CD3^+^ cells with quantitative comparison. T‐SNE plots showing expression levels of G) PD‐L1 and H) CD206 in TAMs with corresponding quantitative comparisons. I) tSNE plots showing FoxP3 expression in CD4+ cells. *p < 0.05, **p < 0.01, *n* = 5.

To investigate the effect of LAA ablation on TME remodeling from a comprehensive perspective, the treated pancreatic tumors were further analyzed via mass cytometry by time‐of‐flight (CyTOF, **Figure** **6**A). Five tumor samples per group, treated with saline or LAA, were analyzed, LAA treatment group showed excellent therapeutic effect as well (Figure 6B). Marker‐based cluster analyses confirmed 12 major immune cell populations, which were further divided into 21 subgroups based on 42 identical markers (Figure 6C,D). Immune patterns of the LAA‐treated tumors significantly differed from the saline‐treated tumors, especially in the distribution of TAMs and CD8^+^ T cells (Figure 6E,F). We observed a substantial decrease of the F4/80^+^ programmed cell death 1 ligand 1 (PD‐L1)^+^ subpopulations (C12), indicating that LAA downregulated the proportion of immunosuppressive macrophages among the total TAM population, because high levels of PD‐L1 contributed to T cell inhibition (Figure 6G). Additionally, LAA attenuated CD206^+^ F4/80^+^ subpopulations (C15), suggesting that LAA boosted the proinflammatory effects, as well as limiting M2‐like TAMs (Figure 6H). Meanwhile, various key factors in the immune microenvironment were also markedly altered. The most interesting change was that LAA treatment significantly reduced the immunosuppressive Treg population (forkhead box P3 (FoxP3)^+^ CD4^+^ T cells), suggesting that LAA contributed to generating an immune‐responsive microenvironment (Figure 6I). Therefore, LAA facilitated TME remodeling toward an immune‐responsive phenotype by increasing CTL infiltration and reprogramming the immunosuppressive components.

## 3 Discussion

The present study demonstrated that LAA is superior to EtOH in both its ablative effect and tumor inhibition in the immunocompetent setting, while LAA and EtOH shared a similar tumor suppression ability in immunodeficient mice. Previous studies only showed that LAA ablation induced higher CD8 expression in the necrotic tissue, but did not ascertain the role of infiltrating T cells, especially CTLs, in enhancing tumor suppression.^[15]^ We found that several key components in the immune response, including DCs, TAMs, and CTLs, substantially influenced the ablation‐related tumor inhibitory outcomes. In particular, LAA treatment altered the antigen presenting ability of DCs, the polarization of TAMs, and the infiltration and cytotoxic function of T cells, which subsequently promoted immune responsiveness, and hence tumor inhibition, on the basis of ablation‐induced necrosis. Our study is the first solid evidence for the vital role of immune regulation in increasing the therapeutic efficacy of chemical ablation.

Our CyTOF analyses also emphasized that LAA caused TME remodeling by significantly changing the immune landscape toward an immune‐responsive phenotype. In contrast to other types of cancer, pancreatic cancer is characterized by an abundance of desmoplastic tissues that prevent infiltration of antitumoral immune cells, consequently generating an immune‐suppressive TME that lacks effective mechanisms of antitumor immunity.^[^ ^36^, ^37^ ^]^ Meanwhile, protumoral immune components, such as Tregs, which work as immune suppressors to markedly promote tumor progression, gradually accumulate in the pancreatic TME, further facilitating immune suppression.^[^ ^38^, ^39^ ^]^ Pancreatic cancer has a markedly impaired immune microenvironment; therefore, it is counted as a “cold” tumor, making it extremely difficult to initialize an immune response in the TME.^[^ ^7^, ^40^ ^]^ The tSNE plots exhibited substantial changes in the immune landscape after LAA ablation, with enhanced infiltration of immune cells and less accumulation of immune‐suppressive components, thus converting the “cold” TME to a “hot” phenotype. Strategies to reverse the TME phenotype have been verified as efficient in many types of solid tumors that were unresponsive to regular immunotherapy.

Contemporary immunotherapeutic approaches mostly rely on regulatory pathways of immune checkpoints and other major co‐stimulatory signals.^[^ ^5^, ^41^ ^]^ Previous studies have shown that the therapeutic effect of current immunotherapy largely depends on the immune phenotypes of the tumors, where “hot” tumors rather than “cold” tumors have a more favorable response to immunotherapy agents.^[^ ^42^ ^]^ Therefore, remodeling the pancreatic cancer TME toward an immune‐responsive phenotype is critical for translational applications of novel immunotherapies. By enhancing immune‐responsive functionality via LAA ablation, we successfully established an immunotherapy‐friendly TME with the potential to augment the immunotherapeutic effect.

## 4 Conclusions

In summary, our study demonstrated a novel mechanism of immune activation by LAA, a type of IL used for chemical ablation, which also features easy delivery and wide diffusion in solid tumors. Our understanding of LAA‐induced immune TME remodeling addresses the vital role of immune regulatory pathways, including DC maturation, macrophage M1 polarization, CTL infiltration, and Treg suppression, in enhancing tumor inhibition after IL ablation. This work proposed a new method, IL ablation, to initiate and amplify the immune response against immune‐suppressive solid tumors, providing a potential solution to immunotherapy inefficiency.

## 5 Experimental Section

### Materials

LAA was synthesized via an ionic metathesis reaction between choline bicarbonate and geranic acid at a molar ratio of 1:1, creating an IL, as previously reported.^[^ ^15^ ^]^ Choline bicarbonate and geranic acid were purchased from Sigma‐Aldrich (St. Louis, MO, USA). Fetal bovine serum (FBS) was purchased from Gibco (Burlington, Canada). Roswell Park Memorial Institute (RPMI) 1640 medium, Dulbecco's modified Eagle's medium (DMEM), McCoy's 5A medium (5A), and penicillin‐streptomycin were purchased from Bristol‐Myers Squibb (Shanghai, China). Membrane tracker CellMask Green and Deep Red were purchased from Thermo Fisher Scientific (Waltham, MA, USA). Hoechst 33342 was purchased from Solarbio (Beijing, China). The reactive oxygen species assay kit and annexin V‐PI apoptosis detection kit were purchased from Beyotime Jiangsu, China). The mouse CD8a+ T cell isolation kit was purchased from Miltenyi Biotec (130‐096‐495, Bergisch Gladbach, Germany). Percoll (17‐0891‐01, GE Healthcare, Chicago, IL, USA), collagenase IV (17104019, Thermo Fisher Scientific), dispase (17105041, Gibco, Grand Island, NY, USA), DNase (D5025, Sigma‐Aldrich), calcium chloride solution (21115, Sigma‐Aldrich), fixation/permeabilization solution kit (555028, BD Biosciences, San Jose, CA, USA), diaminobenzidine (DAB) chromogen kit (BDB2004, Biocare, Redditch, UK) were used. Anti‐mouse CD16/32 antibody (101320), fixation/permeabilization solution kit (555028), leukocyte activation cocktail (550583), anti‐CD86‐PE (553692), anti‐CD45‐BV605 (563053), anti‐CD3‐FITC (555274), anti‐CD49b‐APC (558295), anti‐CD4‐APC‐Cy7 (552051), anti‐CD8‐PE‐Cy7 (552877), anti‐CD69‐BV786 (564683), anti‐PD‐1‐BV421 (562584), anti‐CD45‐BV786 (564225), anti‐MHC II‐BV421 (562564), anti‐CD103‐AF700 (565529), anti‐CD11c‐pc5.5 (560584), and anti‐CD206‐AF647 (565250) antibodies were purchased from BD Biosciences. CFSE (423801), anti‐CD25‐AF700 (102024), and anti‐F4/80‐PE‐Cy7 (157308) antibodies were purchased from Biolegend (San Diego, USA). Alexa Fluor 647 anti‐Calreticulin (ab196159) and anti‐*α*‐SMA (ab7817) antibodies were from Abcam (Cambridge, MA, USA). Antibodies recognizing Ki‐67 (9449), Cleaved Caspase‐3 (9664), and CD8 (98941) were purchased from CST (Danvers, MA, USA). Beyotime provided the anti‐Collagen I antibodies (AF1840).

### In Vitro Imaging System (IVIS) Evaluation of the Ablation Effect

For the subcutaneous model, the KPC cells were injected subcutaneously into the right flank of male C57BL/6 mice at a density of 5 × 10^5^ cells suspended in 50 µL PBS per mouse. For the orthotopic model, C57BL/6 male mice were injected using a sterile insulin needle with 5 × 10^5^ KPC cells suspended in 25 µL mixed medium (Matrigel:PBS = 1:1) at the tail of the pancreas to build an orthotopic tumor model. When tumors reached 1 cm in diameter, the mice were randomly divided into three groups, who received PBS/Cy5 (25 µL), EtOH/Cy5 (25 µL), or LAA/Cy5(25 µL) by intratumor injection. At 24 h after injection, the ablation degree of LAA for subcutaneous and orthotopic models were measured using IVIS. The accurate calculation of the ablation area was completed using Image J (NIH, Bethesda, MD, USA).

### Tumor Permeability of LAA In Vitro

To prove the tumor permeability of LAA, KPC and PSC cells were seeded in an 8‐well chamber and grown to 70% density before the assay. The cells were incubated with fresh cell culture medium containing FITC dye in saline, 1% EtOH (EtOH/FITC), or 1% LAA (LAA/FITC) for different times. After incubation, the cells were stained using membrane tracker CellMask Deep Red (2 µg mL^−1^) for 10 min after washing twice with PBS. Finally, the cell nuclei were stained using Hoechst 33342 (10 µg mL^−1^) for 5 min before cell imaging using a LEICA SP8 confocal microscope (Leica, Wetzlar, Germany). FITC channel: excitation (Ex) = 522 nm, emission (Em) = 530 to 540 nm; Deep Red CellMask channel: Ex = 649 nm, Em = 665 to 675 nm; Blue Hoechst 33342 channel: Ex = 405 nm, Em = 430 to 470 nm. To further prove the tumor permeability of LAA, 5 × 10^2^ KPC cells were seeded in a 96‐well chamber, 7 days later, KPC spheroids were constructed. The KPC spheroids were incubated with fresh cell culture medium containing FITC dye in saline, 1% EtOH (EtOH/FITC), or 1% LAA (LAA/FITC) for 2 h.

### In Vitro ROS Assay

The cell‐permeable ROS sensor dichlorodihydrofluorescein diacetate (DCFH‐DA) was used to detect ROS generation of LAA in KPC cells. 1 × 10^4^ KPC cells were seeded in an 8‐well chamber and cultured overnight. Then, the cells were incubated with a fresh medium containing 1% LAA, 1% EtOH, or saline. After 24 h of culture, the cells were washed with PBS followed by DCFH‐DA (10 × 10^−6^ m) staining at 37 °C for 15 min and Hoechst 33342 staining for 5 min before cell imaging under the LEICA SP8 confocal microscope.

### In Vitro PI Assay

1 × 10^4^ KPC cells were seeded in an 8‐well chamber and cultured overnight. Then, the cells were incubated with a fresh medium containing 1% LAA, 1% EtOH, or saline. After 24 h of culture, the cells were washed with PBS followed by PI staining at 37 °C for 15 min and Hoechst 33342 staining for 5 min before cell imaging under the LEICA SP8 confocal microscope.

### In Vitro ATP Release Assay

ATP release levels were detected using an ATP bioluminescent assay kit (Beyotime, S0026). KPC cells were seeded into 6‐well plates at a density of 1 × 10^5^ per well and cultured overnight. Then, the cells were incubated with a fresh medium containing 1% LAA, 1% EtOH, or saline. After 24 h of culture, the cell supernatant was collected and its ATP content was quantitatively determined using the ATP bioluminescent assay kit according to the manufacturer's instructions.

### In Vitro HMGB1 Release Assay

HMGB1 release levels were detected using an HMGB1 enzyme‐linked immunosorbent assay (ELISA) kit according to the manufacturer's instructions. KPC cells were seeded into 6‐well plates at a density of 1 × 10^5^ per well and cultured overnight. Then, the cells were incubated with fresh medium containing 1% LAA, 1% EtOH, or saline. After 24 h of culture, the cell supernatant was collected and HMGB1 was quantitatively determined using the above‐mentioned ELISA kit.

### In Vitro Calreticulin Exposure Analysis

KPC cells were seeded into 6‐well plates at a density of 1 × 10^5^ per well and cultured overnight. Then, the cells were incubated with fresh medium containing 1% LAA, 1% EtOH, or saline. After 24 h of culture, cells were collected and stained with Alexa Fluor 647 anti‐Calreticulin antibodies (ab196159, Abcam) for 30 min before flow cytometry analysis.

### In Vitro Co‐Culture Assay

Necrotic KPC cells induced by LAA, BMDCs, mouse BMDMs, and mouse spleen‐derived T lymphocytes were co‐cultured to mimic the TME. To analyze DC maturation, BMDCs were co‐cultured with LAA‐induced cells, EtOH‐induced cells, and control cells at a ratio 1:1 for 48 h. Then, the levels of CD86 and MHC II in BMDCs were detected using flow cytometry. To analyze macrophage polarization, BMDMs were co‐cultured with LAA‐induced cells, EtOH‐induced cells, and control cells at a ratio 1:1 for 48 h. Then, the levels of CD86 and MHC II in BMDCs were detected using flow cytometry. To analyze phagocytosis of activated macrophages, KPC cells were labeled with CFSE. Then, the CFSE^+^ KPC cells were cultured with BMDMs in the LAA, EtOH, and saline groups, separately at the ratio of 1:1 for 2 h. Thereafter, the percentage of CFSE^+^ BMDM cells was measured using flow cytometry. To analyze T cell proliferation and maturation, T lymphocytes were incubated with different groups of KPC cells for 48 h in the presence of BMDMs and BMDCs. Then, the levels of CD69, CD103, TNF‐*α*, perforin, and granzyme B were detected using flow cytometry.

### Tumor Immune Microenvironment Assay using Flow Cytometry

Scissors were used to mechanically dissociate the samples into small pieces, which were placed in a plate supplemented with 2% FBS, collagenase IV (1 mg mL^−1^, 17104019, Thermo Fisher Scientific), DNase (10 µg mL^−1^, D5025, Sigma‐Aldrich), Dispase (0.6 mg mL^−1^, 17105041, Gibco), and CaCl_2_ (3 × 10^−3^ m, 21115, Sigma‐Aldrich) in DMEM. The plates were incubated at 37 °C with shaking at 200 rpm for 60 min. Digestion was terminated by the addition of RPMI containing 10% FBS, filtration of the isolated tissue through a 70 µm cell strainer (08‐771‐1, Thermo Fisher Scientific), and washing with PBS once. The cells were resuspended in 36% Percoll solution (GE Healthcare) and separated using density gradient centrifugation to collect immune cells. The immune cells were blocked using TruStain FcX (anti‐mouse CD16/32 antibody) and then stained for cell surface epitopes on ice. If intracellular epitope staining was required, the cells were fixed and then permeabilized using a fixation/permeabilization solution kit (555028, BD Biosciences), followed by staining of intracellular epitopes. Finally, the cells were analyzed by flow cytometry using an LSRFortessa Cell Analyzer (BD Biosciences). These data were analyzed using FlowJo software (TreeStar, Ashland, OR, USA).

### Mass Cytometry (CyTOF) Analysis of the TME

Immune cells were collected from KPC tumors using the method detailed in Section *Tumor Immune Microenvironment Assay using Flow Cytometry*. Immune cells were labeled using antibodies with metal tags. The mixed antibody panel consisted of 42 antibodies conjugated with different metals (Table S1, Supporting Information). The metal signals were detected to evaluate the expression of the conjugated target molecule using the CyTOF system (Helios, Fluidigm, San Francisco, CA, USA).

### Immunohistochemistry (IHC)

CD8, Ki‐67, *α*‐SMA, and Collagen I were labeled using IHC. Tumors harvested at the end of the animal experiments were fixed in 10% buffered formalin and then transected to expose the treatment area. Tissues were subsequently embedded in paraffin. Paraffin‐embedded blocks were sliced into 4 µm sections, placed on positively charged glass slides, baked for 90 min at 68 °C, and then deparaffinized. Antigen retrieval was performed in 10 × 10^−3^ m sodium citrate, pH 6.0 solution, at 100 °C for 10 min and then cooled at room temperature over 30 min. Endogenous peroxidase activity was quenched using 0.3% H_2_O_2_ in 60% methanol for 30 min at room temperature. The sections were blocked in 3% bovine serum albumin for 30 min at room temperature, incubated overnight with primary antibodies at 4 °C, followed by incubation with a biotin‐conjugated secondary antibody for 50 min at room temperature. Target proteins were visualized using a DAB chromogen kit (BDB2004, Biocare) under a light microscope. Tissue sections were then counterstained with hematoxylin. Finally, the slides were dried and covered with mounting medium. Representative images of each tumor were captured using ImageScope software (Leica Biosystems).

### Statistical Analysis

The results were presented as the mean ± standard error. The data were analyzed using GraphPad Prism 7 software (GraphPad Inc., La Jolla, CA, USA). Differences among groups were determined using one‐way ANOVA or Student's *t*‐test. A *p*‐value less than 0.05 was considered statistically significant (*p*‐value: *p < 0.05, **p < 0.01, and ***p < 0.001).

## Conflict of Interest

The authors declare no conflict of interest.

## Author Contributions

J.H. and M.W. contributed equally to this work. J.H. and M.W. performed biochemistry, cell biology, and animal studies, and wrote the manuscript. F.Z. and Z.Y. performed biochemistry studies. S.S. and X.Z. performed animal studies. Q.H. and T.L. conceived, designed, and supervised the project. All authors read and approved the final version of manuscript.

## Supporting information

Supporting Information

## Data Availability

The data that support the findings of this study are available from the corresponding author upon reasonable request.
