# Supplementary material for: An Ionic Liquid Ablation Agent for Local Ablation and Immune Activation in Pancreatic Cancer
Source: Adv Sci (Weinh). 2023 Jan 25;10(10):2206756. doi: 10.1002/advs.202206756 (PMC10074093; doi:10.1002/advs.202206756)
Supplement: Supplementary file 1 — Supporting Information [file ADVS-10-2206756-s001.pdf]

Supporting Information

An ionic liquid ablation agent for local ablation and immune activation in pancreatic cancer

Junming Huang [a, †], Meng Wang[a, †], Fu Zhang[a], Shiyi Shao[a,b], Zhuo Yao[a], Xinyu Zhao[a], Qida Hu[a,b, *], Tingbo Liang[a,b,c,d,e,f, *]

Supplementary Methods

Instruments.

The main instruments used in this project were as follows: IVIS Spectrum Imaging System (Caliper Life Sciences, USA), LEICA SP8 confocal microscope (Leica, GER), LSRFFortessa Cell Analyzer (BD Biosciences, USA), CyTOF Helios system (Fluidigm, USA), Optical microscope (Leica, GER).

Cell culture.

The KPC cell line, derived from spontaneous tumors in a KrasLSL-G12D; Trp53LSL-R172H; Pdx1-Cre mouse model, was a kind gift from the laboratory of Prof. Raghu Kalluri (MD Anderson Cancer Center, Houston, TX, USA). Luciferase- expressing KPC cell line (KPC-Luc) was isolated from KPC cell transfected with firefly luciferase report gene. Pancreatic stellate cell line (PSC) was obtained from American Type Culture Collection (ATCC). The KPC cells were cultured in McCoy's 5A (Modified) Medium (Thermo Fisher Scientific) supplemented with 10% FBS (Biological Industries 04-001-1) and 1% Penicillin/Streptomycin (Cienry CR-15140) in a humidified cell incubator with 5 % CO_2. PSC were cultured in Stellate Cell Medium.

Isolation of BMDM and BMDC from C57BL/6 mouse.

After sacrificing the mouse, we disinfect the skin of the mouse with 70% alcohol and remove the skin and muscles from the legs down to the hip bone, cut the epiphyses of the bones and flush the marrow into a 15 mL centrifuge tube using a 1 mL syringe. The resulting cells were lysed with red blood cell lysis buffer (Beyotime Institute of Biotechnology) and filtered through nylon mesh

filters (70 μm). Centrifuge at 600g and 4°C for 5 min. Dilute the cells at a concentration of 1 × 10^6 cells/mL, add M-CSF at a final concentration of 20 ng/mL (BMDM) or 10 ng/ mL GM-CSF and 5 ng/mL IL- 4 (BMDC), and seed 10 mL per 10 cm cell culture-treated petri dish. Incubate at 37 °C with 5% CO_2 for 3 days. Replace the medium and incubate for another 3 days.

Isolation $CD8^+$ T cells.

$CD8^+$ T cells were isolated from the spleen of C57BL/6 mouse using a $CD8^+$ T cell isolation kit, an LS Column and a MidiMACSTM Separator. Briefly, Spleen was mechanically dissociated into small pieces using a grind rod. The digested tissues were milled into single cells using a 70-μm cell strainer and washed with PBS for 3 times. Red blood cell lysis buffer was used to remove red blood cells. Then the $CD8^+$ T cell were purified using a mice $CD8^+$ T cell isolation kit according the manufacturer's instructions.

Establishment of pancreatic cancer model.

Male nude mice (4-6 weeks) and Male C57BL/6 mice (4-6 weeks) were purchased from the Model Animal Research Center of Nanjing University, China. All animals were maintained in a pathogen-free environment under controlled humidity and temperature. The animal experiments were performed in accordance with the China Animal Protection Law. Animal suffering was minimized or prevented at all time to improve their welfare. Nude and C57BL/6 mice were subcutaneously inoculated with 2 × 10^5 KPC cancer cells in subcutaneous model experiment of pancreatic cancer. Pancreatic tail of C57BL/6 mice were inoculated with 2 × 10^5 KPC cancer cells in orthotopic model experiment of pancreatic cancer. 25 μL 25% LAA was injected intratumorally in all animal experiments.

***In vivo* Biocompatibility assay.**

At the end of the LAA treatment in vivo, the peripheral blood (1mL per mouse) was collected under anesthesia for blood biochemistry detection. The biochemistry indicators including Alanine Transaminase (ALT), Aspartate Transaminase (AST), UREA, Creatinine (CREA), were the liver and kidney function index. The liver and kidney function tests were performed to investigate the biocompatibility of the LAA treatment.

And the major organs (hearts, livers, spleens, lungs and kidneys) of the mice were collected to evaluate the toxicity of the LAA treatment by H&E staining. For the H&E assay, the organs were fixed in 10% neutral buffered formalin for 24 h. After paraffin embedding, they were cut into 4

μm-thick sections and deparaffinized after being baked at 68°C for 90 min. The sections were dehydrated in graded ethanol and immersed in hematoxylin staining solution for 5 min. The color of the sections changed from blue to red 2 seconds after adding 50 uL 1% hydrochloric acid ethanol. The sections were then immersed in eosin staining solution for 5 min and washed with ddH_2O. Finally, the representative images were captured by ImageScope software.

CyTOF analysis.

CyTOF experiment and analysis are supported by Zhejiang Puluoting Health Technology Co. Ltd. The information of antibody was showed in **Table S1.**

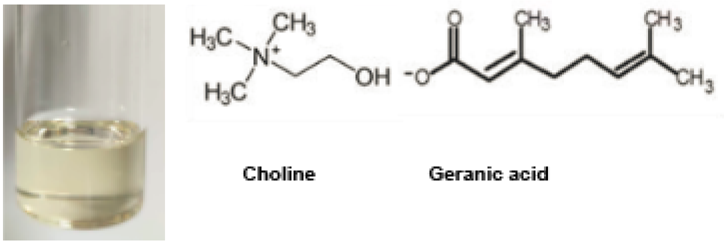

Figure S1. Photograph of neat ionic liquid synthesized by choline and geranic acid.

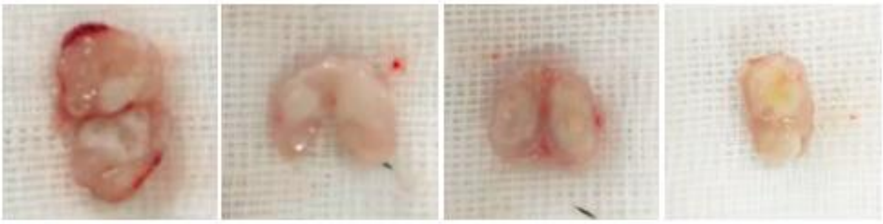

5%LAA 10%LAA 25%LAA 50%LAA

Figure S2. Images of C57BL/6 mice orthotopic pancreatic cancer model at 24 hours following injection of 5%, 10%, 25% or 50% LAA.

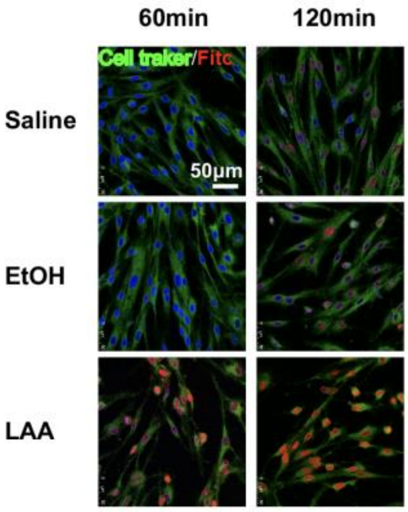

Figure S3. Confocal images of PSC cells uptaking Dox (5μM) supported by EtOH (1%) and LAA (1%) at 60 min and 120 min after treatment (green, cell membrane; and red, Dox). Scale bars, 50μm.

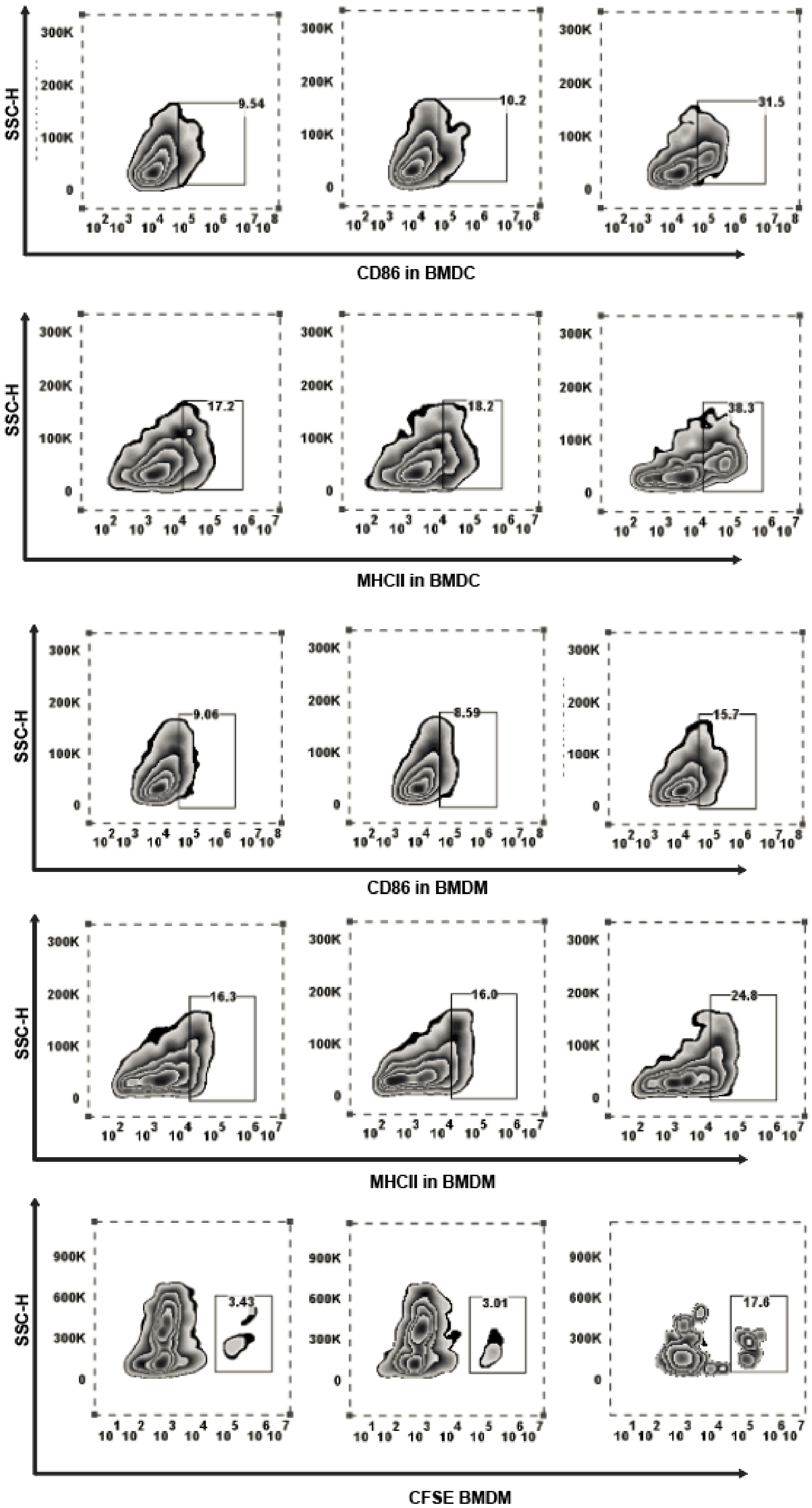

Figure S4. The representative cytometry patterns shown related to **Figure 3F and 3G**.

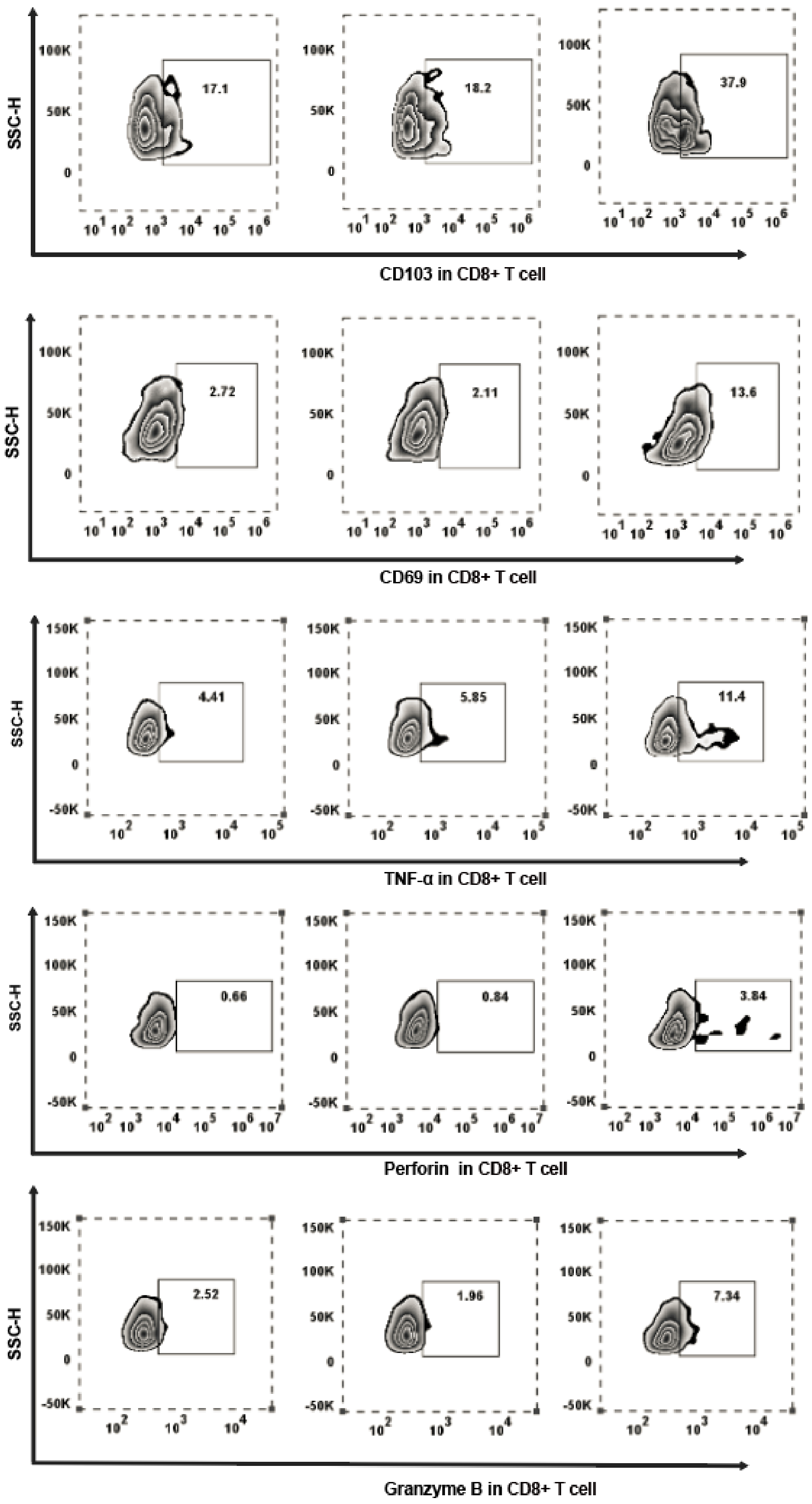

Figure S5. The representative cytometry patterns shown related to **Figure 3H**.

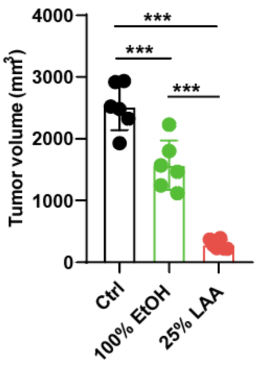

Figure S6. Comparison of the final tumor volumes in different groups.

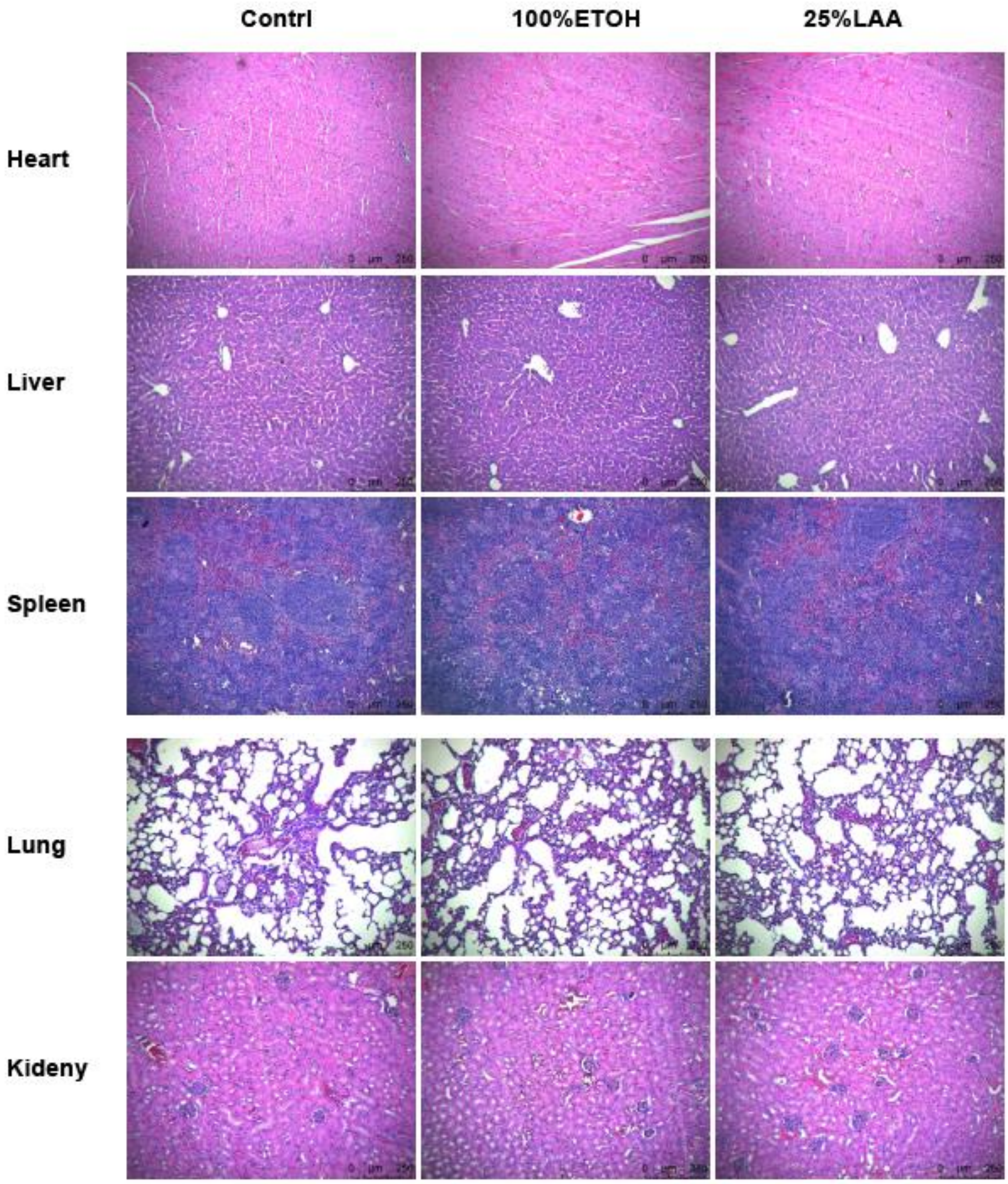

Figure S7. Pathology studies of the organs, including heart, liver, spleen, lung and kidney in C57BL/6 mice treated with Saline, 100%EtOH and 25%LAA.

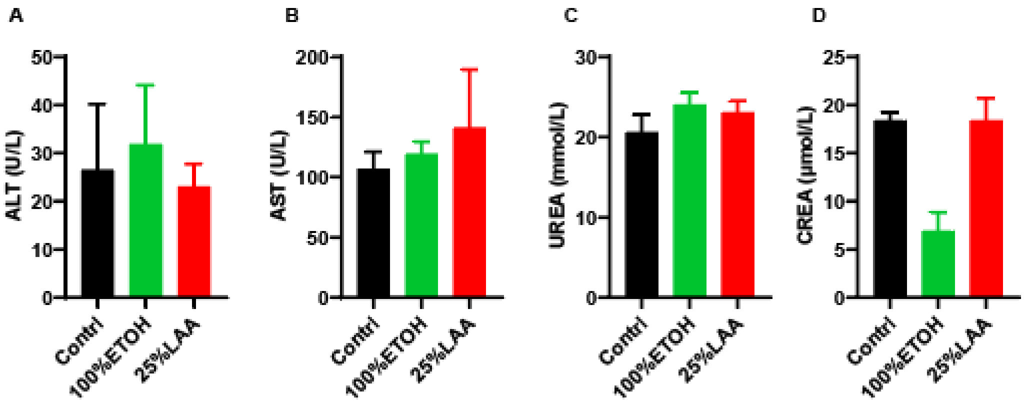

Figure S8. Results of the serum biochemical index in C57BL/6 mice treated with PBS, 100%EtOH and 25%LAA.

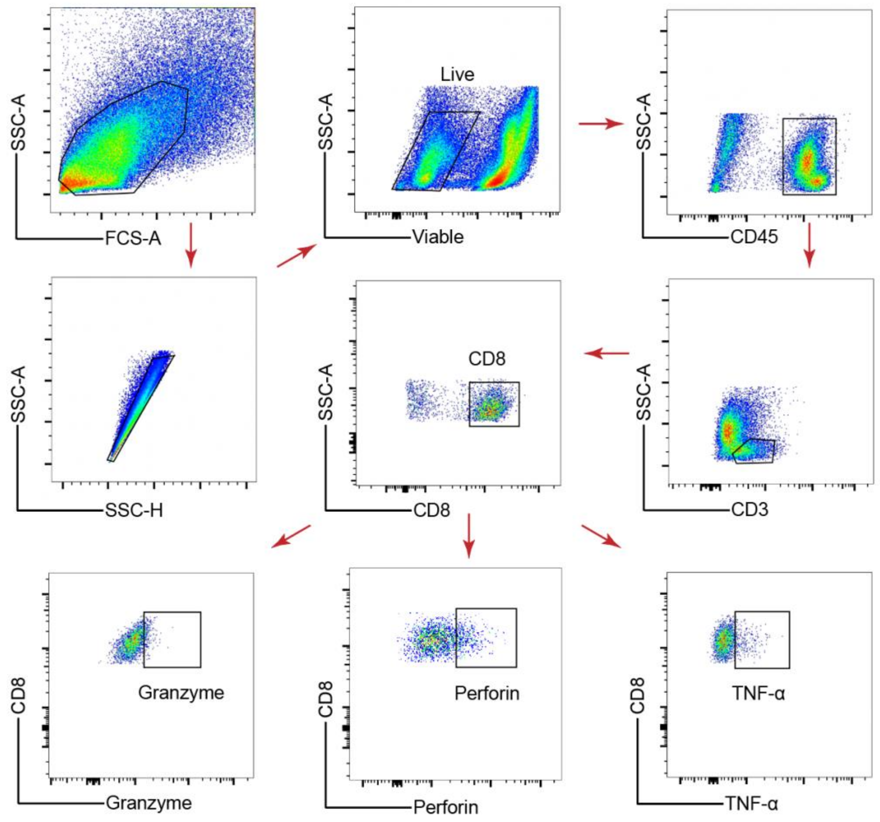

Figure S9. Gating strategy of FACS analysis for detecting Granzyme B, TNF-α and Perforin expression of CD8^{+} T cells in the TEM of orthotopic pancreatic cancer.

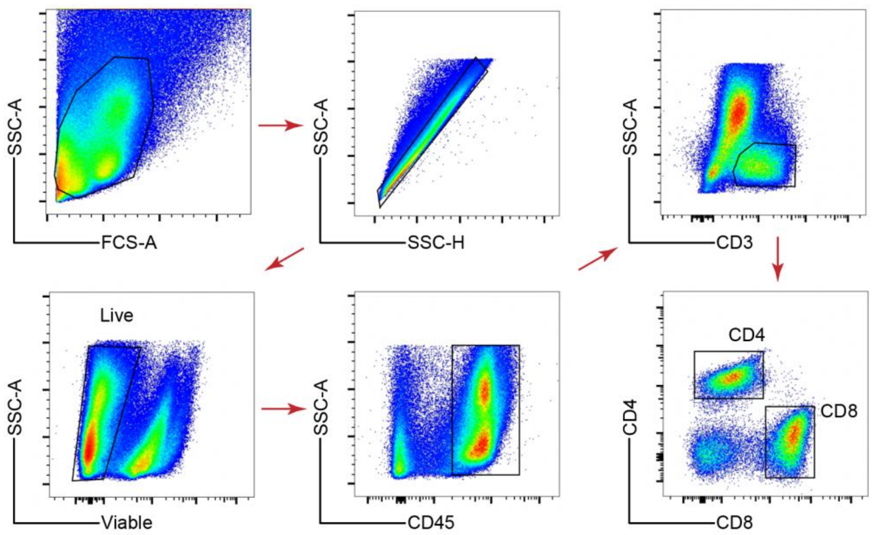

\

Figure S10. Gating strategy of FACS analysis for detecting $CD4^{+}$ and $CD8^{+}$ T cells in the TEM of orthotopic pancreatic cancer.

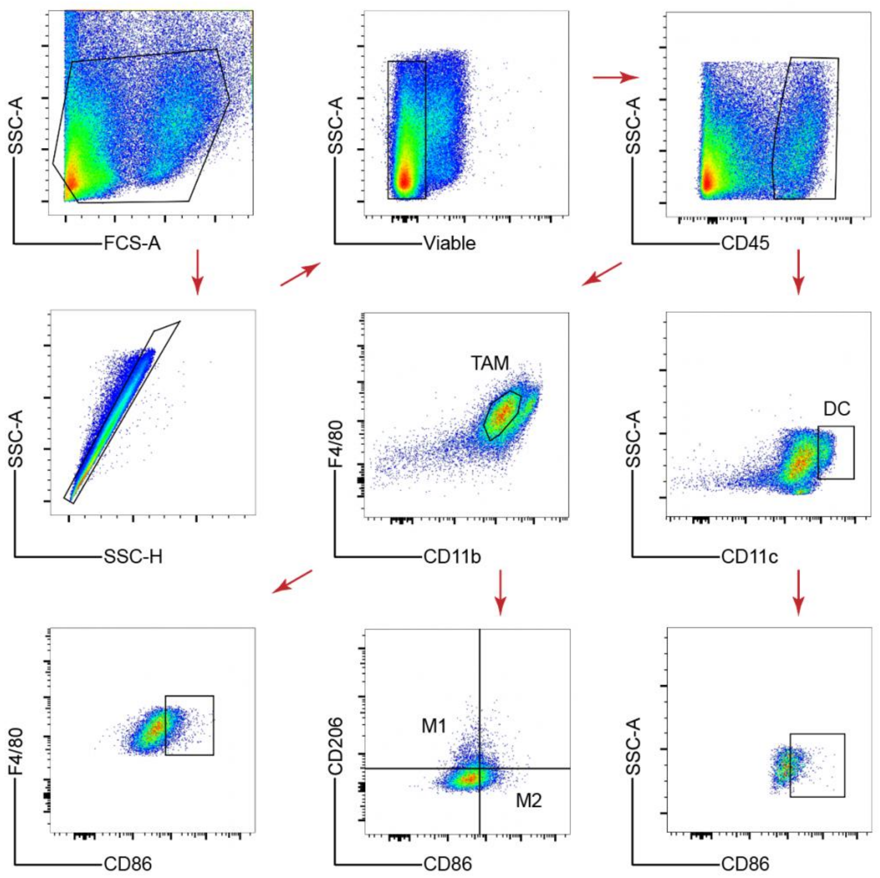

Figure S11. Gating strategy of FACS analysis for detecting the expression of CD86 and CD206 in TAM and CD86 in DCs in the TEM of orthotopic pancreatic cancer.

Table S1: List of Antibodies in CyTOF

List	Channel	Epitope	Brand	Category number
1	89Y	CD45	Biolegend	103102
2	115In	CD3e	Biolegend	100302
3	139La	Ki67	eBioscience	14-5698-82
4	141Pr	CD24	Biolegend	101802
5	142Nd	MHC II	Biolegend	107602
6	143Nd	CD45R_B220	Biolegend	103202
7	144Nd	CD183_CXCR3	Biolegend	126502
8	145Nd	CD163	Biolegend	155302
9	146Nd	CD279_PD1	Biolegend	135202
10	147Sm	CD80	Biolegend	104702
11	148Nd	Ly6C	Biolegend	128002
12	149Sm	CD19	Biolegend	115502
13	150Nd	IL10	Biolegend	505002
14	151Eu	CD62L	Biolegend	104402
15	152Sm	CD11c	Biolegend	117302
16	153Eu	CD44	Biolegend	103002
17	154Sm	Tbet	BioLegend	644802
18	155Gd	CD223_Lag3	Biolegend	125202
19	156Gd	CD14	Biolegend	123302
20	157Gd	CD366_Tim3	Biolegend	119702
21	158Gd	TCR γ/δ	Biolegend	118140
22	159Tb	F4/80	BioRAD	MCA497G
23	160Gd	CD86	Biolegend	105002
24	161Dy	CD274_PDL1	Biolegend	124302
25	162Dy	CD69	Biolegend	104502
26	163Dy	CD25	Biolegend	101902

27	164Dy	RORγ	R&D	MAB6109
28	165Ho	IFNγ	Bio-Xcell	BE0055
29	166Er	Ly6G	Biolegend	127602
30	167Er	CD206	Biolegend	141702
31	168Er	FoxP3	eBioscience	14-5773-82
32	169Tm	CD127_IL7Ra	Biolegend	135002
33	170Er	CD161_NK1.1	Biolegend	108702
34	171Yb	GATA3	eBioscience	14-9966-82
35	172Yb	Perforin	Fluidigm	3172018B
36	173Yb	Granzyme B	Fluidigm	3173006B
37	174Yb	CD152_CTLA4	Biolegend	106302
38	175Lu	IL2	Biolegend	503802
39	176Yb	TNFα	Biolegend	506302
40	197Au	CD4	Biolegend	100520
41	198Pt	CD8a	Biolegend	100746
42	209Bi	CD11b	BioLegend	101202
